# Existe um Papel para a Ultrassonografia Pulmonar no Prognóstico de Pacientes com Insuficiência Cardíaca?

**DOI:** 10.36660/abc.20201283

**Published:** 2021-03-03

**Affiliations:** 1 Universidade de São Paulo Instituto do Coração do Hospital das Clínicas Faculdade de Medicina São PauloSP Brasil Instituto do Coração do Hospital das Clínicas da Faculdade de Medicina da Universidade de São Paulo, São Paulo, SP – Brasil

**Keywords:** Insuficiência Cardíaca, Prognóstico, Hospitalização, Mortalidade, Pulmão/ultrassonografia, Peptídeos Natriuréticos

As hospitalizações por insuficiência cardíaca (IC) são mais comumente motivadas por congestão sistêmica e pulmonar, levando a uma taxa de mortalidade de 5–15% e a uma taxa de reinternação de até 50% em 90 dias.^1^ Embora a meta usual durante a hospitalização seja a descongestão completa, 30% dos pacientes apresentam congestão residual quando da alta.^2^ Sinais de congestão, altas pressões de enchimento e níveis elevados de peptídeos natriuréticos estão associados a maiores taxas de mortalidade e reinternação, e a congestão residual na alta também está associada a pior prognóstico.^3^^,^^4^

A avaliação da congestão geralmente é difícil devido à baixa sensibilidade e/ou especificidade dos resultados da anamnese.^1^ A radiografia de tórax e os peptídeos natriuréticos podem melhorar a avaliação, mas a ultrassonografia pulmonar (USP) tem sido usada recentemente como uma ferramenta sensível nesse cenário.^5^ Ela pode estimar a pressão atrial direita usando o diâmetro e a variação da veia cava e avaliar a congestão pulmonar por meio da contagem de linhas B nas zonas torácicas. As linhas B são artefatos hiperecoicos no USP que aparecem como linhas verticais da linha pleural até a parte inferior da tela e representam o espessamento dos septos interlobulares. Dados recentes relatam a associação de linhas B na USP com maiores taxas de mortalidade e hospitalização.^6^

Neste número dos Arquivos Brasileiros de Cardiologia, uma revisão sistemática e metanálise avaliou o valor prognóstico da congestão pulmonar representada pelas linhas B em pacientes com IC.^7^ A metanálise incluiu 8 estudos envolvendo pacientes hospitalizados e ambulatoriais e três análises diferentes foram executadas. Primeiro, entre pacientes com IC hospitalizados, >15 e >30 linhas B na alta se correlacionaram significativamente com risco aumentado de óbito ou hospitalização por IC em três estudos (FC, 3,37, IC 95%, 1,52–7,47; I^2^=0% p=0,003 e FC, 4,01, IC 95% 2,29–7,01; I^2^=0%, p<0,001, respectivamente). Segundo, as linhas B na alta estiveram associadas a maior risco de hospitalização em dois outros estudos (FC, 1,05, IC 95% 1,01–1,09, I^2^=87%, p=0,01), com maior associação e menor heterogeneidade na análise de subgrupo considerando as linhas B>30 na alta (FC, 9,01, IC 95%, 2,08–28,93, I^2^=0%, p<0,001). Terceiro, entre pacientes ambulatoriais, as linhas B>3 aumentaram significativamente o risco de óbito ou hospitalização por IC em cinco estudos (FC, 3,21, IC 95%, 2,09–4,93, I^2^=10%, p<0,00001).

Algumas limitações devem ser destacadas: a heterogeneidade dos estudos não permitiu aos autores definir um único desfecho primário para incluir todos os estudos, limitando, assim, o número de pacientes para cada análise. Existem diferentes protocolos para avaliação das linhas B e o manejo de pacientes com IC não foi avaliado. Nenhum dos estudos estratificou a IC por fração de ejeção, embora a fração de ejeção média de todos os estudos fosse inferior a 50%. Grandes ensaios clínicos randomizados são necessários para confirmar esses achados.

Por outro lado, essa metanálise nos permite formular uma hipótese. A avaliação da congestão na alta costuma ser falha, mas, ao mesmo tempo, a descongestão é fundamental.^8^ Portanto, uma ferramenta extra poderia ser usada para ajudar na descongestão. Essa foi a justificativa para o ensaio PRIMA II, um ensaio randomizado que avaliou a porção N-terminal do pró-hormônio do peptídeo natriurético tipo B como alvo para dar alta a pacientes com IC descompensada aguda, mas nenhuma diferença foi encontrada em óbitos ou hospitalizações em 180 dias.^9^ Mas quais seriam os efeitos da avaliação da USP na alta visando um determinado número de linhas B? Ajudaria a orientar a terapia para a descongestão completa? Em última análise, reduziria as hospitalizações e a mortalidade na IC? Definitivamente, mais dados são necessários, e este é um campo promissor.
