# Supplementary material for: Existe um Papel para a Ultrassonografia Pulmonar no Prognóstico de Pacientes com Insuficiência Cardíaca?
Source: Arq Bras Cardiol. 2021 Mar 3;116(3):393–4. [Article in Portuguese] doi: 10.36660/abc.20201283 (PMC8159545; doi:10.36660/abc.20201283)
Supplement: Translation

# Translation: Is There a Role for Lung Ultrasonography in the Prognosis of Heart Failure Patients?

**Affiliations:** 1 São PauloSP Brazil Instituto do Coração do Hospital das Clínicas da Faculdade de Medicina da Universidade de São Paulo, São Paulo, SP – Brazil

**Keywords:** Heart Failure, Prognosis, Hospitalization, Mortality, Lung/ultrasonography, Natriuretic Peptides

Heart failure (HF) hospitalizations are most commonly motivated by systemic and pulmonary congestion, leading to 5–15% of mortality rate and up to 50% of readmission rate in 90 days.^1^ Although the usual goal during hospitalization is complete decongestion, 30% of patients have residual congestion at discharge.^2^ Signs of congestion, high filling pressures and elevated natriuretic peptides are associated with higher mortality and readmission rates, and residual congestion at discharge is also associated with worse prognosis.^3^^,^^4^

Congestion assessment is usually difficult given the low sensitivity and/or specificity of physical examination findings.^1^ Chest radiography and natriuretic peptides can improve evaluation, but lung ultrasonography (LUS) has been recently used as a sensitive tool in this scenario.^5^ It can estimate right atrial pressure using vena cava diameter and variation and evaluate lung congestion by counting B-lines in chest zones. B-lines are hyperechoic artefacts on LUS which appear as vertical lines from the pleural line to the bottom of the screen and represent thickening of interlobular septa. Recent data report association of B-lines in LUS with higher mortality and hospitalization rates.^6^

In this issue of Arquivos Brasileiros de Cardiologia, a systematic review and metanalysis evaluated the prognostic value of pulmonary congestion represented by B-lines in patients with HF.^7^ The metanalysis included 8 studies involving hospitalized and outpatients and three different analyses were executed. Firstly, among hospitalized HF patients, >15 and >30 B-lines at discharge significantly correlated with increased risk of death or HF hospitalization in three studies (HR, 3.37, 95% CI, 1.52–7.47; I^2^=0% p=0.003 and HR, 4.01, 95% IC 2.29–7.01; I^2^=0%, p<0.001, respectively). Secondly, B-lines at discharge were associated with increased risk of hospitalization in two other studies (HR, 1.05, 95% CI 1.01–1.09, I^2^=87%, p=0.01), with higher association and lower heterogeneity in subgroup analysis considering B-lines>30 at discharge (HR, 9.01, 95% CI, 2.08–28.93, I^2^=0%, p<0.001). Thirdly, among outpatients, B-lines>3 significantly increased risk of death or HF hospitalization in five studies (HR, 3.21, 95% IC, 2.09–4.93, I^2^=10%, p<0.00001).

Some limitations must be highlighted: the heterogeneity of studies did not allow the authors to define a single primary outcome to include all studies, thus limiting the number of patients for each analysis. There are different protocols for B-line evaluation, and management of HF patients was not evaluated. None of the studies stratified HF by ejection fraction, although the mean ejection fraction of all studies was below 50%. Large randomized trials are required to confirm these findings.

On the other hand, this metanalysis enables us to formulate a hypothesis. Congestion evaluation at discharge is often flawed, but, at the same time, decongestion is fundamental.^8^ Therefore, an extra tool could be used to help achieving decongestion. This was the rationale for the PRIMA II trial, a randomized trial evaluating N-terminal pro-B-type natriuretic peptide as a target to discharge acute decompensated HF patients, but no difference was found in death or hospitalizations in 180 days.^9^ But what would be the effects of LUS evaluation at discharge targeting a certain number of B-lines? Would it help guiding therapy towards complete decongestion? Ultimately, would it reduce hospitalizations and mortality in HF? Definitely, more data is needed, and this is a promising field.
